# Competing endogenous RNAs in lung cancer

**DOI:** 10.20892/j.issn.2095-3941.2020.0203

**Published:** 2021-02-15

**Authors:** Meilian Zhao, Jianguo Feng, Liling Tang

**Affiliations:** 1 Key Laboratory of Biorheological Science and Technology, Ministry of Education, College of Bioengineering, Chongqing University, Chongqing 400044, China; 2 Department of Anesthesiology, The Affiliated Hospital of Southwest Medical University, Luzhou 646000, China

**Keywords:** CeRNA, lung cancer, biological functions, biomarker

## Abstract

Competing endogenous RNAs (ceRNAs) containing microRNA response elements can competitively interact with microRNA via miRNA response elements, which can combine non-coding RNAs with protein-coding RNAs through complex ceRNA networks. CeRNAs include non-coding RNAs (long non-coding RNAs, circular RNAs, and transcribed pseudogenes) and protein-coding RNAs (mRNAs). Molecular interactions in ceRNA networks can coordinate many biological processes; however, they may also lead to ceRNA network imbalance and thus contribute to cancer occurrence when disturbed. Recent studies indicate that many dysregulated RNAs derived from lung cancer may function as ceRNAs to regulate multitudinous biological functions for lung cancer, including tumor cell proliferation, apoptosis, growth, invasion, migration, and metastasis. This study therefore reviewed the research progress in the field of non-coding and protein-coding RNAs as ceRNAs in lung cancer, and highlighted validated ceRNAs involved in biological lung cancer functions. Furthermore, the roles of ceRNAs as novel prognostic and diagnostic biomarkers were also discussed. Interpreting the involvement of ceRNAs networks in lung cancer will provide new insight into cancer pathogenesis and treatment strategies.

## Introduction

Lung cancer has the highest mortality rate of all cancers globally, accounting for about 10%–20% of total cancer deaths. Due to high metastasis, the 5 year survival is approximately 18%. Lung cancer is a molecularly heterogeneous cancer and understanding its biology is crucial for the development of effective therapies. The World Health Organization divides lung cancer into two main types based on its biological characteristics and clinical treatment practices: small cell lung cancer (SCLC) and non-small cell lung cancer (NSCLC)^1^. The most common NSCLCs are lung adenocarcinoma (LUAD) and lung squamous carcinoma (LUSC). The proportion of NSCLC in all lung cancers is 80%–85%. NSCLC is the leading cause of cancer-related death, causing about 1.6 million deaths since 2012^2^. Lung cancer is similar to most malignancies in that it has distinct molecular characteristics, and is composed of subpopulations of cells, or clones, resulting in intratumoral heterogeneity^3^. LUAD is the most heterogeneous and aggressive among lung cancer subtypes, and has a very high tumor mutation burden associated with increased postsurgical relapse possible in treated patients^4^. This indicates that there is a greater metastases propensity during early tumor development related to increased intratumoral heterogeneity. SCLC accounts for approximately 14% of all lung cancers, and has some of the most representative clinical characteristics and distinctive malignancies in the entire field of oncology. SCLC is an aggressive high grade malignant cancer associated with a high growth rate and widespread metastases during early tumor development, which contributes to the extremely poor prognoses of cancer patients^3^. Currently, the primary treatment methods for lung cancer include surgery, chemotherapy, radiation, and targeted therapy. Therapeutic approach options depend on several factors, including the cancer grade and stage^5^. In spite of the progress made in diagnosing and treating tumors in the past 25 years, patients have a low survival due to the failure and/or limitations of chemotherapy^6,7^. This problem forces people to constantly seek new diagnoses and therapy strategies. Therefore, in-depth institutional research and novel therapeutic target discovery are presently the major emphases of cancer research.

With the development of high-throughput sequencing and constant updating of algorithms, large amounts of non-encoded RNAs have been found to perform multitudinous biological functions in humans, and are involved in the generation and development of tumors^4^. MicroRNAs (miRNAs) are a family of regulatory, endogenously expressed, small non-coding RNA molecules that control mRNA expression, primarily by binding to the 3′-untranslated regions (3′-UTRs)^8^. MiRNAs play a vital role as tumor suppressors or oncogenes by silencing or activating target gene expression, which has differential expression patterns in cancer^9^. There are more than 500 miRNA genes that can recognize and specifically bind their targets through miRNA response elements (MREs) in the human genome. MREs are normally located in coding sequences, especially in the 3′-UTRs of various types of RNAs, such as circRNAs, mRNAs, lncRNAs, and transcribed pseudogenes^10^. In theory, any RNA transcriptome containing miRNA-binding sites has the ability to combine specifically with miRNAs, which can act as ceRNAs transcribers, including protein-coding mRNAs and non-coding RNAs (ncRNA). For example, long non-coding RNAs transcribe pseudogenes, and circular RNAs can compete with protein-coding mRNAs to bind shared miRNAs by acting as ceRNAs or natural microRNA sponges. Post-transcriptional regulators are important for gene expression^11–13^. The ceRNA studies have revealed a new mechanism for RNA-RNA interactions, communication, and co-regulation, in which miRNAs can affect gene expression by binding to mRNAs. Additionally, ceRNAs can competitively interact with miRNAs via MREs. For example, Kumar et al.^14^ discovered a novel protein-coding gene, *HMGA2*, that exerts its effects largely independent of its protein coding function. HMGA2 functions as a ceRNA for the let-7 miRNA family to promote lung cancer progression. Therefore, it is critical and significant to recognize and elucidate the regulatory mechanisms of lung cancer ceRNA networks and their impacts on cancer diagnosis, prognosis, and treatment, which in turn has an impact on human development and disease.

In this review, we focused on research progress involving non-coding and protein-coding RNAs as ceRNAs in lung cancer, and highlighted validated ceRNAs involved in lung cancer biological functions. Furthermore, the role of ceRNAs as novel prognosis and diagnosis biomarkers was also discussed.

## The ceRNA crosstalk in lung cancer

The ceRNA hypothesis was formally proposed by Salmena et al.^15^ in 2011. It describes a previously unknown large-scale regulatory mechanism regulating gene expression via transcriptomes. MiRNAs not only affect the transcription and stability of RNAs at the post-transcriptional level through binding to target genes, but RNAs in turn can also influence miRNAs^15–17^. This original novel pattern is named the “RNA→miRNA→RNA” interplay. The miRNAs act as negative or positive regulators of gene translation, that decrease/enhance the stability of target RNAs or limit/promote their expression efficiencies and levels. RNA transcripts contain the same MREs on their 3′-UTRs, which can act as ceRNAs to communicate and regulate their expression levels by competitively binding the same sequences of miRNAs^18^. The conditions needed for the formation of a ceRNA crosstalk mechanism remain unknown. Recent studies have shown that the effect of ceRNA activity depends on a series of factors, including the relative concentration of miRNAs/ceRNAs, changes of RNA 3′-UTRs, RNA editing, and RNA binding proteins (RBPs)^13,19^. First, the relative abundances of the ceRNAs and the target miRNAs are clearly important, because too much or too little ceRNA or target miRNA will reduce ceRNA-miRNA competition efficiency^19^. Second, the combination effectiveness of a ceRNA for microRNA molecules may be decided by its subcellular localization and interaction with RBPs^12,20^. If any of these factors are altered, ceRNA network imbalance can occur, and thus contribute to cancer occurrence and development. Recent studies have shown that an increasing number of ceRNAs were found through bioinformatics technology, which plays an important role in lung cancer studies. Interpreting the ceRNA network as it pertains to cancer can therefore provide new insights into cancer pathogenesis and therapeutic strategies.

### The lncRNAs as ceRNAs

There are about 20,000 protein-coding genes, which only account for < 2% of the total genome sequence; nevertheless, at least 90% of genes are actively transcribed into ncRNA. This implies that lncRNAs can play significant regulatory roles in biological processes. The lncRNAs are a type of ncRNA molecule defined by a lack of protein-coding potential (often determined computationally) and are commonly defined as being longer than 200 nucleotides. Evidence suggests that lncRNAs are aberrantly expressed in various types of cancer cells and contribute to the initiation and development of several common hallmarks of cancer^21–23^. In general, lncRNAs can regulate gene expression levels at different stages and through different pathways, including chromatin modification, transcription, and post-transcription. For chromatin modification, lncRNAs induce chromatin formation in a specific genomic locus by interacting with chromatin remodeling complexes, resulting in decreased gene expression. Moreover, lncRNAs regulate promoters or interact with RNA-binding proteins and transcriptional factors to modulate gene transcription levels. Based on these features, lncRNAs are involved in many important biological phenomena, such as gene imprinting, transcriptional enhancement, chromosome looping, and antisense regulation^24^. The lncRNAs exert regulatory functions as ceRNAs by competitively binding to shared sequences of miRNAs and influencing the expression levels of their downstream target genes (mRNA). The lncRNAs can form lncRNA-miRNA-mRNA interactions, which are called ceRNA networks. Recent studies revealed that lncRNAs act as ceRNAs to participate in lung cancer development by competing with miRNAs and protein-coding mRNAs to modulate biological functions. The lncRNA-mediated competitive RNA crosstalk in lung cancer progression has been identified and is shown in **Table 1**.

**Table 1:** Overview of long noncoding RNAs as competing endogenous RNAs in lung cancer

CeRNA	Target miRNA	mRNA	Expression	Key factors and pathways	Biological functions	Tumor types	CeRNA role	References
LINC01123	MiR-199a-5p	C-myc	Upregulated	–	Proliferation	NSCLC	Oncogene	^25^
Linc00173	MiR-218	Etk	Upregulated	β-catenin signaling	Proliferation, migration-invasion	SCLC	Oncogene	^26^
HOTAIR	MiR-214-3p	PDPK1	Upregulated	–	Growth	NSCLC	Oncogene	^27^
TTN-AS1	MiR-142-5p	CDK5	Upregulated	EMT( TWIST1, Twist, Snail and ZEB1)	Proliferation, migration and invasion	LUAD	Oncogene	^28^
PVT1	MiR-199a-5p	HIF1α	Upregulated	–	Migration and proliferation	NSCLC	Oncogene	^29^
LINC00336	MIR6852	CBS	Upregulated	P53 pathway	Proliferation	NSCLC	Oncogene	^30^
LCAT1	MiR-4715-5p	RAC1	Upregulated	–	Proliferation, migration and invasion	Lung cancer	Oncogene	^31^
TINCR	MiR-544a	FBXW7	Downregulated	–	Proliferation and invasion	Lung cancer	Suppressor	^32^
NNT-AS1	MiR-129-5p	NNT-AS1	Upregulated	–	Proliferation, migration and invasion	NSCLC	Oncogene	^33^
UCA1	MiR-193a	HMGB1	Upregulated	–	Proliferation and migration	Lung cancer	Oncogene	^34^
H19	MicroRNA-107	NF1	Upregulated	–	Proliferation and migration	NSCLC	Oncogene	^35^
PVT1-5	MiR-126	SLC7A5	Upregulated	–	Proliferation	Lung cancer	Oncogene	^36^
XLOC_008466	MiR-874	MMP2/XIAP	Upregulated	–	Proliferation, apoptosis and invasion	NSCLC	Oncogene	^37^
HOTTIP	MiR-574-5p	EZH1	Upregulated	–	Proliferation	SCLC	Oncogene	^38^
LINC01234	MiR-140	OTUB1	Upregulated	SP1	Proliferation, metastasis and apoptosis	NSCLC	Oncogene	^39^
LINC00858	MiR-422a	KLK4	Upregulated	–	Proliferation	NSCLC	Oncogene	^40^
SNHG7	MiR-193b	FAIM2	Upregulated	E-cadherin, N-cadherin	Proliferation, apoptosis and metastasis	NSCLC	Oncogene	^41^
ZEB1-AS1	MiR-409-3p	ZEB1	Upregulated	Caspase 3, Bax and Bcl-2	Proliferation, apoptosis	NSCLC	Oncogene	^42^
MALAT1	MiR-124	STAT3	Upregulated	STAT3	Proliferation, colony formation and apoptosis	NSCLC	Oncogene	^43^
H19	MiR-17	STAT3	Upregulated	STAT3	Growth, migration and invasion	NSCLC	Oncogene	^44^
NR2F2-AS1	MiR-320b	BMI1	Upregulated	–	Apoptosis, Proliferation and invasion	NSCLC	Oncogene	^45^
OGFRP1	MiR-124-3p-	LYPD3	Upregulated	EMT, caspase-9 and caspase-3	Proliferation, apoptosis, migration and invasion	NSCLC	Oncogene	^46^
PTCH1	MiR-101-3p	SLC39A6	Upregulated	Hh pathway, Vimentin, N-cadherin (CDH2)	Migration, invasion and adhesion	NSCLC	Oncogene	^47^
NEAT1	MiR-377-3p	E2F3	Upregulated	P57, p21 and Bcl2	Growth, apoptosis, migration and invasion	NSCLC	Oncogene	^48^
HOXA11-AS	MiR-454-3p	Stat3	Upregulated	Cleaved-caspase-3/9, EMT (Snail, Twist)	Proliferation, apoptosis, metastasis	LUAD	Oncogene	^49^
PRNCR1	MiR-448	HEY2	Upregulated	EMT	Proliferation, invasion and migration	NSCLC	Oncogene	^50^
LncRNA 1308	MiR-124	ADAM 15	Upregulated	–	Proliferation, apoptosis, invasion and migration	NSCLC	Oncogene	^51^
FLVCR1-AS1	MiR-573	E2F3	Upregulated	–	Proliferation, migration and invasion	NSCLC	Oncogene	^52^
LINC00641	MiR-424-5p	PLSCR4	Downregulated	–	Proliferation, apoptosis and migration	NSCLC	Suppressor	^53^
TP73-AS1	MiR-449a	EZH2	Upregulated	–	Proliferation, growth	NSCLC	Oncogene	^54^
XIST	MiR-137	PXN	Upregulated	–	Invasion	NSCLC	Oncogene	^55^
MYEOV	MiR-30c-2-3p	TGFBR2, USP15	Upregulated	TGF-β/SMAD pathway	Invasion and metastasis	NSCLC	Oncogene	^56^
NEAT1	Mir-193a-3p	USF1	Upregulated	Caspase-3/7	Proliferation, apoptosis, invasion and migration	LUAD	Oncogene	^57^
LINC00702	MiR-510	PTEN	Downregulated	PTEN pathway, AKT pathway	Proliferation, apoptosis, invasion	NSCLC	Suppressor	^58^
MALAT1	MiR-200b	ZEB1, E2F3	Upregulated	EMT, TFAP2C, ZEB1, E2F3	Proliferation, apoptosis and metastasis	LUAD	Oncogene	^59^
HOXD-AS1	MiR-147a	PRB	Upregulated	–	Growth, proliferation, apoptosis	NSCLC	Oncogene	^60^
DANCR	MiR-138	Sox4	Upregulated	EMT	Proliferation, apoptosis, migration, invasion	NSCLC	Oncogene	^61^
DGCR5	MiR-211-5p	EPHB6	Downregulated	–	Growth, migration and invasion	NSCLC	Suppressor	^62^
SNHG20	MiR-154	ZEB2, RUNX2	Upregulated	–	Proliferation, migration and invasion, apoptosis, growth	NSCLC	Oncogene	^63^
XIST	MiR-367/141	ZEB2	Upregulated	TGF-β induced EMT	Migration and invasion	NSCLC	Oncogene	^64^
LIN28B	Let-7	HMGA2	Upregulated	TGFBR3	Migration and invasion	NSCLC	Oncogene	^65^
DLX6-AS1	MiR-144	PRR11	Upregulated	–	Proliferation, apoptosis, invasion and migration	NSCLC	Oncogene	^66^
SBF2-AS1	MiR-338-3p/ 362-3p	E2F1	Upregulated	Cyclin D1, p21	Proliferation	LUAD	Oncogene	^67^
SNHG6	MiR-26a-5p	E2F7	Upregulated	EMT	Proliferation, migration and invasion	LUAD	Oncogene	^68^
Linc00668	MiR-147a	Slug	Upregulated	EMT	Proliferation, migration and invasion	NSCLC	Oncogene	^69^
DANCR	MiR-496	mTOR	Upregulated	mTOR pathway	Proliferation, migration and invasion, apoptosis, growth	LUAD	Oncogene	^70^
HMMR-AS1	MiR-138	SIRT6	Upregulated	–	Apoptosis, Proliferation	LUAD	Oncogene	^71^
GMDS-AS1	MiR-96-5p	CYLD	Upregulated	Bax, Bcl-2 and PCNA	Proliferation, apoptosis	LUAD	Suppressor	^72^
LINC00667	MiR-143-3p	RRM2	Downregulated	CDK2 and p-AKT	Growth, Proliferation	NSCLC	Oncogene	^73^
Linc00673	MiR-150-5p	ZEB1	Upregulated	TGFB1/Smad, EMT (Snail, ZEB1)	Apoptosis, Proliferation, migration and invasion	NSCLC	Oncogene	^74^
SNHG1	MiR-145-5p	MTDH	Upregulated	EMT, TGF-β	Proliferation, migration and invasion	NSCLC	Oncogene	^75^
NEAT1	Let-7a	IGF-2	Upregulated	P53, p21, EMT, IGF-2 signaling pathway	Apoptosis, Proliferation, invasion and migration, growth	NSCLC	Oncogene	^76^
NEAT1	MiR-377-3p	E2F3	Upregulated	P21 p57	Growth, Proliferation	NSCLC	Oncogene	^77^
LINC81507	MiR-199b-5p	CAV1	Downregulated	STAT3 pathway	Proliferation, metastasis, migration and invasion	NSCLC	Suppressor	^78^
NORAD	MiR-136-5p	E2F1	Upregulated	–	Proliferation	NSCLC	Oncogene	^79^
MINCR	MiR-126	SLC7A5	Upregulated	–	Proliferation , migration, apoptosis, growth	NSCLC	Oncogene	^80^
JPX	MiR-145-5p	Cyclin D2	Upregulated	CCND2	Proliferation, colony formation and migration	NSCLC	Oncogene	^81^
MEG3	MicroRNA-7-5p	BRCA1	Downregulated	Bcl-2 and Bax	Apoptosis	NSCLC	Suppressor	^82^
NEAT1	MiR-98-5p	MAPK6	Upregulated	BCL-2 , MMP-9, MMP-2	Growth, migration and invasion	NSCLC	Oncogene	^83^
XIST	Let-7i	BAG-1	Upregulated	–	Apoptosis, Proliferation	LUAD	Oncogene	^84^
PVT1	MiR-216b	Beclin-1	Upregulated	BCL2, Bax	Apoptosis	NSCLC	Oncogene	^85^
LINC00707	MiR-145	MRP1	Upregulated	Bcl-2 and Bax	Apoptosis	NSCLC	Oncogene	^86^
XIST	MiR-374a	LARP1	Upregulated	P-STAT3, cyclin D1	Proliferation, migration and invasion	NSCLC	Oncogene	^87^
LINC00485	MiR-195	CHEK1	Upregulated	Bax, Bcl-2, VEGF, HIF-1α	Proliferation, apoptosis	LUAD	Oncogene	^88^
Linc00665	MiR-98	AKR1B10	Upregulated	EMT, Bcl-2, Bax, ERK, p-ERK and SP1	Proliferation, migration, invasion, apoptosis, growth	LUAD	Oncogene	^89^
HOTTIP	MiR-216a	BCL-2	Upregulated	BCL-2	Apoptosis	SCLC	Oncogene	^90^
XIST	MiR-449a	Bcl-2	Upregulated	PARP-1, caspase-9 and Bcl-2	Growth, apoptosis, migration and invasion	NSCLC	Oncogene	^91^
ZFAS1	MiR-150-5p	HMGA2	Upregulated	–	Proliferation, invasion, apoptosis	NSCLC	Oncogene	^92^

### The circRNA as ceRNAs

Evidence presented in 1993 indicates that junctions of mis-spliced ets-1 exons lead to the formation of circular RNA (circRNA) molecules, which is the first case of circular transcripts being processed from nuclear pre-mRNA in eukaryotes^93^. The circRNAs are defined as a large class of non-coding RNAs that are generated by canonical splicing machinery; an upstream splice site is covalently linked to a downstream splice site during backsplicing. The circRNAs are closed and have no open linear tails, making them insensitive to exonucleases. This suggests that circRNAs are very stable, which might reflect their important non-coding functions. In addition, circRNAs have been related to and participated in many human diseases, including diabetes mellitus, neurological disorders, chronic inflammatory diseases, cardiovascular diseases, and cancer^94–97^. In addition, they may accumulate during the onset of illness. CircRNAs are abundant and evolutionarily conserved. Additionally, some circRNAs exert important biological functions by acting as ceRNA, which contain many miRNA competing binding sites to regulate protein synthesis and function. Hence, circRNAs exert tumor suppressive or oncogenic functions by acting as miRNA sponges to combine multiple diverse miRNAs, rather than a particular miRNA. Higher circRNA expression levels may therefore cooperatively function to sponge numerous miRNAs and affect the progression of lung cancer (**Table 2**).

**Table 2:** Overview of circular (circ)RNAs as competing endogenous RNAs in lung cancer

CeRNA	Target MiRNA	mRNA	Expression	Key factors and pathways	Biological functions	Tumor types	CeRNA role	References
Circ_0020123	MiR-488-3p	ADAM9	Upregulated	–	Apoptosis, migration and invasion	NSCLC	Oncogene	^98^
Circ-FOXM1	MiR-1304-5p	PPDPF, MACC1	Upregulated	–	Proliferation, migration, invasion and apoptosis	NSCLC	Oncogene	^99^
CircHIPK3	MIR124-3p	STK11	Upregulated	MIR124-3p-STAT3-PRKAA/AMPKα	Proliferation, migration, invasion	NSCLC	Oncogene	^100^
CircTP63	MiR-873-3p	FOXM1	Upregulated	FOXM1, CENPA and CENPB	Proliferation, growth	LUSC	Oncogene	^101^
VANGL1	MiR-195	Bcl-2	Upregulated	Bcl-2 and Bax	Proliferation, apoptosis, migration and invasion	NSCLC	Oncogene	^102^
Circ_0003645	MiR-1179	TMEM14A	Upregulated	–	Migration, invasion, growth, apoptosis	NSCLC	Oncogene	^103^
Circ-ENO1	MiR-22-3p	ENO1	Upregulated	Cleaved-caspase 3/6/9/PARP, EMT	Migration, invasion, apoptosis, growth	LUAD	Oncogene	^104^
CircPTK2	MiR-429/200b-3p	TIF1γ	Downregulated	TGFB1/Smad, EMT	Metastasis, migration, invasion	NSCLC	Suppressor	^105^
CircRNA100146	MiR-361-3p/615-5p	SF3	Upregulated	PCNA, p53, NFAT5, COL1A1, TRAF3	Proliferation, apoptosis, growth, invasion	NSCLC	Oncogene	^106^
CircPVT1	MiR-497	Bcl-2	Upregulated	Bcl-2 and Bax	Proliferation, apoptosis, growth	NSCLC	Oncogene	^107^
CircPVT1	MiR-125b	E2F2	Upregulated	E2F2 Signaling, cyclin D, Rb, c-Fos	Proliferation, apoptosis, migration and invasion	NSCLC	Oncogene	^108^
HOXA11-AS	MiR-124	Sp1	Upregulated	Ecadherin, vimentin, β-catenin, Snail and Slug	Proliferation, invasion	NSCLC	Oncogene	^109^
Circ_0074027	MiR-185-3p	BRD4/MADD	Upregulated	BRD4 and MADD	Proliferation, apoptosis, migration and invasion	NSCLC	Oncogene	^110^
Circ-CMPK1	MiR-302e	Cyclin D1	Upregulated	Cyclin D1	Proliferation	NSCLC	Oncogene	^111^
CESRP1	MiR-93-5p	Smad7/p21	Downregulated	P21, p-Smad2/3, Smad4 and Smad7	Migration and invasion	SCLC	Suppressor	^112^
CircARHGAP10	MiR-150-5p	GLUT1	Upregulated	E-cadherin, N-cadherin and Snail	Proliferation, migration	NSCLC	Oncogene	^113^

### Pseudogenes as ceRNAs

Pseudogenes, defined as dysfunctional transcripts of protein-coding genes, represent a particular type of lncRNA once thought to be “genomic junk,” and were considered without any biological function^114,115^. However, recent improvements of the genome project have revealed that many pseudogenes have transcriptional activities. Furthermore, pseudogenes can exert a positive effect because they are stable. However, their dysregulation can contribute to the occurrence of diseases, and their disordered expressions contribute to the development and progression of multiple cancers^116^. Increasing evidence has shown that pseudogenes play key roles in tumor suppression or oncogenesis. Despite the abundance of pseudogenes identified in human cancers or other diseases, the pathophysiological roles of pseudogenes in lung cancer remain poorly understood.

### The mRNAs as ceRNAs

Researchers currently are very interested in the effects of non-coding RNAs. Nevertheless, coding transcripts (mRNA) are more abundant and show the highest conservation of miRNA binding sites. Previous studies have shown that the particular 3′-UTR of a mRNA could act as a ceRNA to regulate the activity of endogenous miRNAs, which then may affect the expression levels of downstream mRNAs^117,118^. Recent studies also showed that mRNAs serve as miRNA sponges, which exert the most influence during lung cancer progression (**Table 3**).

**Table 3:** Overview of mRNAs as competing endogenous RNAs in lung cancer

CeRNA	Target miRNA	mRNA	Expression	Key factors and pathways	Biological functions	Tumor types	CeRNA role	References
AEG-1	MiR-30a	Vimentin/N-cadherin/Snail/E-cadherin	Upregulatede	EMT, TGF-β and TNF-α	Migration and invasion	NSCLC	Oncogene	^119^
PCNX	MiR-26/182/340/506	Skp2	Upregulatede	EGF-induced Pakt	Growth, proliferation and apoptosis	Lung cancer	Oncogene	^120^
ZEB1	MiR-181b	ITGA1, AC9	Upregulatede	ITGA1 and AC9	Migration and metastasis	LUAD	Oncogene	^121^
HMGA2	Let-7	Tgfbr3	Upregulatede	TGF-β signalling	Growth, invasion	NSCLC	Oncogene	^14^
FOXO1	MiR-96	DUSP1	Upregulatede	EGFR/EGF signaling pathway, MMP2 and MMP11	Growth, proliferation, migration and invasion	NSCLC	Oncogene	^122^

–: not reported.

## The ceRNAs function in lung tumor signaling pathways and biological processes

In recent years, the important roles of ceRNA interactions during cancer initiation and progression processes have been accepted. An increasing number of ceRNAs has been reported to be involved in the cellular signaling pathways of lung cancer, such as in β-catenin signaling, the PI3K/AKT signaling pathway, the PTEN pathway, the IGF-2 signaling pathway, Wnt signaling, the STAT3/p-STAT3 pathway, and transforming growth factor-β (TGF-β) signaling (**Tables 1–3**). Furthermore, ceRNAs competing with miRNAs are involved in the biological functions of lung cancer, such as in tumor cell proliferation, apoptosis, growth, cell cycle, invasion, migration, and metastasis (**Tables 1–3**). Some ceRNAs have also been found to be dysregulated in lung tumor tissues, playing tumor-oncogenic or tumor-suppressor roles. In this review, we therefore mainly discussed validated ceRNAs and their biological functions in lung cancer.

### The ceRNAs and the signaling pathways involved in lung cancer cell proliferation

Cellular metabolism is the basis of all biological activities^123^. Compared with normal differentiated cells, most cancer cells rely primarily on aerobic glycolysis to generate a large amount of energy during cellular processes. Cell growth and proliferation must be regulated^124,125^. In addition, the capacity of unlimited multiplication is a typical characteristic of tumor cells. Cell proliferation requires the accumulation of an intracellular biomass, such as proteins and lipids. It is therefore essential for cellular replication and division to produce proteins, lipids, and nucleic acids. Notably, cancer is characterized by abnormal proliferation resulting from aberrant expression of various cell cycle proteins; therefore, cell cycle regulators are considered to be vital factors in tumor proliferation^126^. Studies have reported that ceRNAs are involved in lung tumor proliferation.

**Figure 1:**
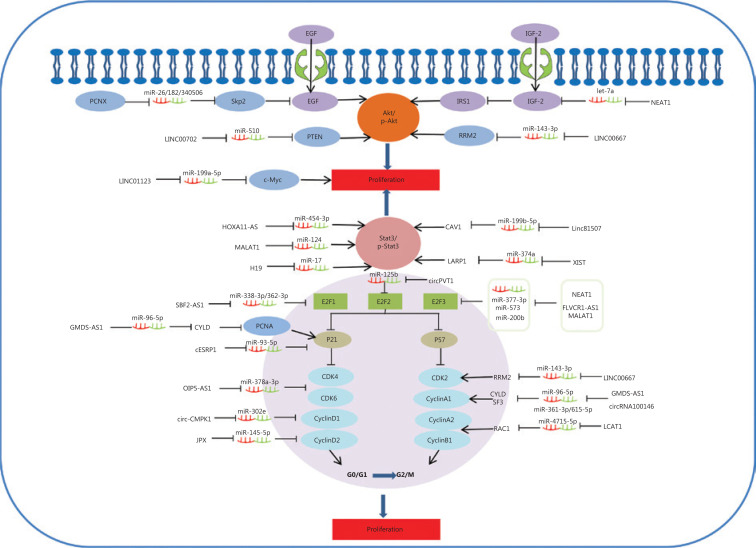
Competing endogenous RNAs (CeRNAs) and the signaling pathways involved in lung cancer cell proliferation. The AKT pathway and STAT3/p-STAT3 signaling pathways play an important role in the proliferation of lung cancer cells; PCNX, NEAT1, LINC00702, LINC00667, HOXA11-AS, MALAT1, H19, Linc81507, and XIST function as ceRNAs and are involved in these signaling pathways. In addition, the cell cycle is considered an important factor in tumor proliferation. SBF2-AS1, GMDS-AS1, cESRP1, OIP5-AS1, circ-CMPK1, JPX, circPVT1, NEAT1, LINC00667, circ-100146, and LCAT1 act as ceRNAs to regulate cell proliferation by targeting cell cycle regulators.

STAT3 functions as a significant protein in the JAK-STAT signaling pathway, and has been identified as having a significant role in tumorigenesis^127,128^. In addition, STAT3/p-STAT3 plays an important role in regulating multiple cell processes, such as cell proliferation^129^. Some lncRNAs function as ceRNAs to competitively bind shared sequences of miRNAs and regulate mRNA generation and function, which has been reported to participate in cell proliferation via inactivating the STAT3 signaling pathway in lung cancer (**Figure 1**)^43,44,49,78,87^. LINC81507 is decreased in NSCLC and acts as a ceRNA to sponge miR-199b-5p by regulating the CAV1/STAT3 signaling pathway. This suggests that LINC81507 serves as a tumor suppressor for cell proliferation in NSCLC^78^.

It is well-known that Akt kinase is commonly activated in human cancers^130–132^. Some ceRNAs impact cell proliferation by targeting the protein kinase B (AKT) signaling pathway and β-catenin signaling during lung cancer progression^26,58,73,120^. Skp2, as a unique E3 ligase of Akt, has been reported to trigger K63-linked Akt ubiquitination and to also be involved in Akt phosphorylation and activation in response to EGF stimulation^133,134^. The EGF-induced Akt phosphorylation affected PCNX, which functions as a ceRNA to target oncogenic Skp2^120^. Research results also indicate that Linc00702 may act as a ceRNA for miR-510, and may inhibit proliferation of NSCLC cells via activating PTEN, while PTEN’s downstream target, p-AKT is significantly altered^58^. IGFs (IGF1 and IGF2) are capable of promoting cellular proliferation by stimulating phosphorylation of MAPK and Akt. IRS1 is the substrate for the IGF receptor, and phosphorylation of IRS1 is mediated by IGF-2 to subsequently activate the Akt signaling pathway^134,135^. LncRNA NEAT1 acts as a let-7a sponge to facilitate proliferation via the IGF2/AKT/MAPK signaling pathway in NSCLC (**Figure 1**)^76^.

Cell cycle regulators are considered important factors in tumor proliferation. Some circRNAs regulate cell proliferation by targeting cell cycle regulators. P21 is a well-known inhibitor of cell cycle progression, which can arrest cells in G1/S and G2/M phases by inhibiting CDK4,6/cyclin-D and CDK2/cyclin-E (cell cycle-related proteins), respectively^136–138^. It is believed that p21 acts by suppression of E2F (E2F1, E2F2, and E2F3) activity to regulate cell growth. In brief, p21 interacts with these factors and disrupts their interactions to inhibit the cell cycle and cell proliferation progression. SBF2-AS1, a lncRNA upregulated in NSCLC, could increase E2F1 expression through competitively binding with miR-338-3p/miR-362-3p. During tumor proliferation, this results in decreased *p21* gene expression and increased cyclinD1^67^. E2F3 is a core oncogene involved in promoting NSCLC proliferation progression. However, NEAT1 as a ceRNA for hsa-miR-377-3p, could lead to the derepression and functional incapacitation of its endogenous target, E2F3^48^. Furthermore, E2F3 overexpression reverses the inhibitory efficiency of miR-377-3p in downregulating protein levels of E2F3, CDK4, cyclinD1, and cyclinD2, and also reverses the favorable efficiency of miR-377-3p in the upregulation of p21 and p57 protein levels^48^. Although the functions of E2Fs are most commonly relevant to and involved in cell cycle regulatory mechanisms, studies have shown that non-classical roles for E2Fs beyond proliferation regulation are largely associated with cancer. The activation of all E2F activators (E2F1, E2F2, and E2F3) can lead to uncontrolled proliferation (**Figure 1**)^136^. For example, some lncRNAs, such as NEAT1, FLVCR1-AS1, and MALAT1, function as ceRNAs, which positively regulate E2F3 expression through respectively inhibiting miR-377-3p, miR-573, and miR-200b during NSCLC cell proliferation^48,52,59^. LncRNAs SBF2-AS1 and NORAD regulate E2F1 expression by binding specific miR-338-3p/362-3p or miR1365p, respectively, to promote NSCLC cell proliferation^67,79^. In addition, circRNA circPVT1 enhances proliferation through sponging miR-125b and activating E2F2 signaling in NSCLC^108^. Activation of cyclin-dependent kinases (CDKs) and dysregulation of the cell-cycle mechanism can promote tumor cell-cycle progression, which in turn can stimulate uncontrolled tumor cell proliferation, a key characteristic of cancer^139^. A further investigation showed that OIP5-AS1 functioned as a ceRNA of miR-378a-3p. Its inactivation caused a decrease of proliferation-associated proteins, CDK4 and CDK6, and inhibited tumor cell proliferation in NSCLC cells^140^.

### The ceRNAs and signaling pathways involved in lung cancer cell apoptosis

**Figure 2:**
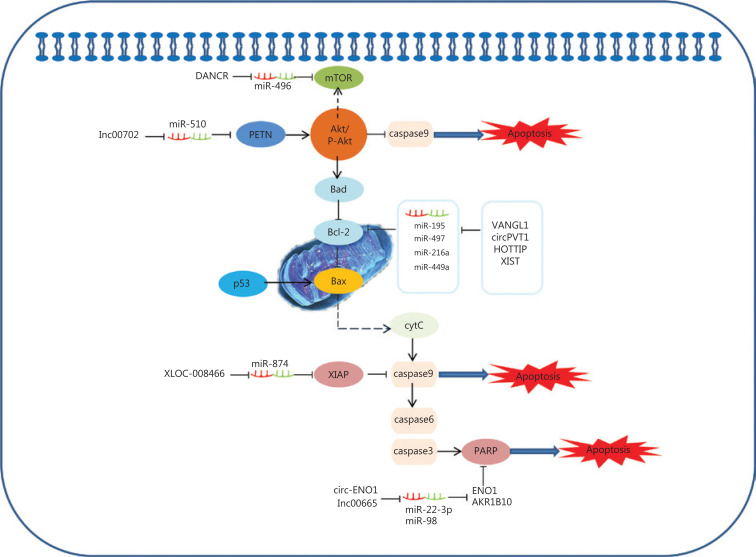
Competing endogenous RNAs (ceRNAs) and the signaling pathways involved in lung cancer cell apoptosis. The BCL-2 protein family of mitochondrial proteins plays a pivotal role in regulating programmed cell death by controlling proapoptotic and anti-apoptotic intracellular signals; VANGL1, circPVT1, HOTTIP and XIST function as miRNA sponges to regulate the expression of apoptosis-related protein Bcl-2. The cytC released from mitochondria promotes caspase9 activation, whereas XIAP blocks the expression of caspase9. Caspase9 then activates procaspase3 and procaspase6, resulting in cell apoptosis. XLOC_008466 functions as an oncogene in non-small cell lung cancer by directly binding to the miR-874-MMP2/XIAP axis to affect cell apoptosis. Both Circ-ENO1 and lnc00665 are upregulated in lung cancer, and act as ceRNAs to prohibit apoptosis by inhibiting PARP.

The ability of cancer cells to suppress apoptosis is critical for carcinogenesis^141^. Recent advances reveal that potential ceRNAs may regulate cell apoptosis, and can thus be used to predict the effects of progression and treatments in response to apoptosis in lung cancer. In mitochondria, the BCL-2 protein family determines the commitment of cells to an intrinsic apoptotic response. This protein family also plays a pivotal role in controlling programmed cell death by regulating intracellular proapoptotic and anti-apoptotic signals^142,143^. There are four characterized ceRNAs, VANGL1, circPVT1, HOTTIP, and XIST, which may function as ceRNAs to regulate the expression level of the apoptosis-related protein, Bcl-2 (**Figure 2**)^90,91,102,107^. Bad inhibits the pro-survival Bcl-2-like proteins, and thereby enables activation of the pro-apoptotic effector Bax, which then disrupts the outer mitochondrial membrane. The cytochrome C (cytC) released from the mitochondria promotes caspase9 activation, whereas E3 ubiquitin-protein ligase XIAP (the X-linked inhibitor of an apoptosis protein) blocks the expression of caspase9^144,145^. Caspase9 can activate procaspase3 and procaspase6, resulting in cell apoptosis. XLOC_008466 functions as an oncogene in NSCLC by directly binding to the miR-874-MMP2/XIAP axis, which indicates that XLOC_008466 may act as a ceRNA to affect cell apoptosis^37^. Both Circ-ENO1 and lnc00665 are upregulated in lung cancer, and act as ceRNAs to prohibit apoptosis. Silencing circ-ENO1 and lnc00665 caused PARP levels to decline, and elevated levels of cleaved-caspase3, cleaved-caspase6, and cleaved-caspase9^89,104^. The tumor suppressor gene, *p53*, is the most frequently mutated gene in all human tumor cells. The p53 tumor-suppressor pathway regulates hundreds of genes that are involved in multiple biological processes, including cell cycle arrest, senescence, and apoptosis^146,147^. Activation of the phosphatidylinositol-3-kinase (PI3-K)/protein kinase B (AKT) pathway is also associated with cell apoptosis. LINC00702 is an upstream regulator of the miR-510-PTEN axis, and acts via activating the Akt signaling pathway to regulate lung cancer apoptosis^58^.

### The ceRNAs and signaling pathways involved in lung cancer cell invasion and migration

**Figure 3:**
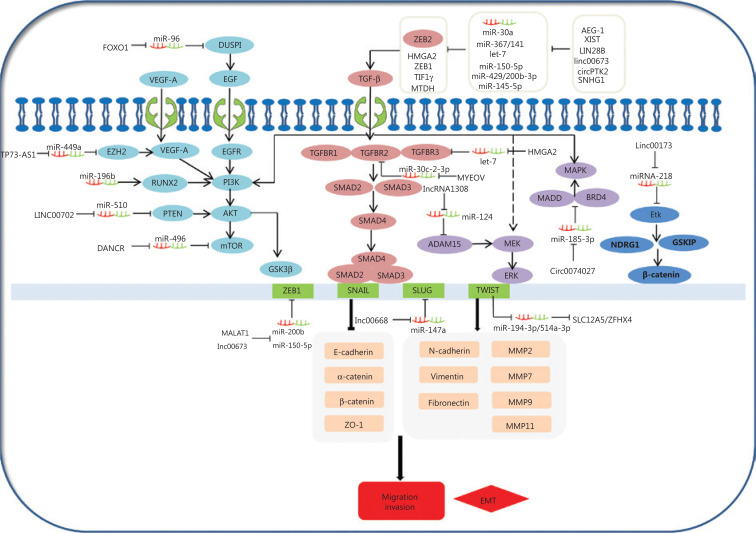
Competing endogenous RNAs (ceRNAs) and the signaling pathways involved in lung cancer cell invasion and migration. The epithelial-mesenchymal transition (EMT) is important in the behavior of cells during cancer progression, which confers tumor cells with migratory and invasive properties. The TGF-β pathway plays a central role in inducing the EMT in all types of tumors. TGFβs specifically bind to complexes of TGF-βR1, TGF-βR2, and TGF-βR3, leading to the phosphorylation of SMAD2 and SMAD3, which then forms complexes with SMAD4. AEG-1, XIST, LIN28B, linc00673, circPTK2, SNHG1, and cESRP1 as ceRNAs affect the signaling/Smad-induced EMT. In addition to Smad-dependent pathways in TGF-β family signaling, the TGF-β also induces the non-Smad TGF-β signaling pathways, such as PI3K/Akt/GSK3β, MEK-ERK, and MAPK pathways. Smad-dependent pathways are involved in TGF-β family signaling. FOXO1, TP73-AS1, LINC00702, DANCR, and circ0074027 act as ceRNAs to participate in non-Smad TGF-β signaling pathways in the invasion and metastasis of lung cancer.

The epithelial-mesenchymal transition (EMT) is a common behavior of cells during cancer progression, and gives tumor cells migratory and invasive properties. The TGF-β pathway plays a central role in inducing the EMT in all types of tumors. TGF-βs can specifically bind to complexes of TGF-β receptor type 1 (TGFβR1), TGF-β receptor type 2 (TGFβR2), and TGF-β receptor type 3 (TGFβR3). This in turn leads to the phosphorylation of SMAD2 and SMAD3, which then form complexes with SMAD4. Moreover, the phosphorylated complexes of SMAD2, SMAD3, and SMAD4 lead to EMT activation related to molecules involved in several different mechanisms. Expressions of EMT-related transcription factors, SNAIL, SLUG, ZEB1, and TWIST, are activated by SMAD phosphorylated complexes. These collaborate to restrain EMT-related marker expressions of E-cadherin, α-catenin, β-catenin, and ZO-1, and in turn, stimulate the expressions of vimentin, N-cadherin, fibronectin, and MMPS. For example, TWIST1, as a ceRNA, can regulate SLC12A5/ZFHX4 levels by sponging miR-194–3p/miR-514a-3p to influence the EMT progression in lung cancer^148^. The linc00668-centered competing endogenous RNA mechanism then promotes invasion and migration of lung cancer by regulating slug expression levels^69^. Additionally, TGFBR2 and TGFBR3 expressions are regulated by the MYEOV and HMGA2 ceRNAs via a TGF-β signaling pathway, which plays a novel role in the invasion and metastasis of NSCLC^14,56^. Furthermore, AEG-1, XIST, LIN28B, linc00673, circPTK2, SNHG1, and cESRP1 function as sponges of miR-30a,, miR-367/141let-7, miR-150-5p, miR-429/miR-200b-3p, miR-145-5p, and miR-93-5p, and indirectly impact the TGF-β/Smad-induced EMT; subsequently, they regulate the molecular mechanism of the EMT-related transcription factors and markers (**Figure 3**)^64,65,74,75,105,112,119^. This suggests that ceRNAs activate EMT-associated regulators through the TGF-β/SMADs signaling pathway.

Diverse signaling pathways play predominant roles in the initiation and progression of the EMT to convert epithelial cells into mesenchymal cells, affecting gene levels and morphology. In addition to its signaling pathway through SMADs, TGF-β also induces PI3K/Akt/GSK3β, MEK-ERK, and MAPK signaling pathways (**Figure 3**), which also induce the EMT. PI3K-AKT is activated through TGF-β, which induces invasion and migration of lung cancer^149,150^. This shows that the PI3K-AKT-GSK3β pathway plays an essential role in the EMT process^151^. Overexpression of miR-196b inhibits the TGF-β induced EMT process, and also suppresses the PI3K-AKT-GSK3β pathway by inactivating Runx2 in lung cancer^152^. Additionally, epidermal growth factor (EGF) and vascular endothelial growth factor (VEGF) may induce the PI3K-AKT pathway, and subsequently increase the EMT-related regulator levels. For example, the TP73-AS1-miR-449a-EZH2 axis ceRNA network promotes tumor migration and metastasis via regulating VEGF-A/AKT signaling in lung cancer^54,153^. Finally, FOXO1, an important transcription factor, serves as a ceRNA to sponge miR-96. FOXO1 can also induce lung cancer migration and invasion progression by affecting the EGFR signaling pathway^122^.

TGF-β can also activate the MEK-ERK and MAPK pathways, leading to the EMT initiation and progression. For example, a novel lncRNA (lncRNA 1308) targets the miR-124/ADAM15, to regulate cell invasion through activation of the MEK-ERK pathway^51,154^. Circ_0074027 induces invasion of lung cancer via activating bromodomain-containing protein 4 (BRD4) and MAPK-activating death domain-containing protein expression levels^110^. Ultimately, loss of linc00173 expression downregulates Etk by functioning as a ceRNA to target miRNA-218, and induces the inactivation of GSKIP and NDRG1. This causes the nuclear translocation of β-catenin and promotes lung cancer migration and invasion^26^.

## The ceRNAs as novel prognoses and diagnoses biomarkers

Tumor (or cancer) biomarkers are biological molecules in cancer patients that may be used to characterize known tumors. These biomarkers are produced by the tumor itself or by the body in response to the tumor, and can be used as an indicator of diagnosis, prognosis, and prediction. Most importantly, they can be used to provide information about the patient’s survival or response to therapy. The ultimate objective is to acquire available diagnostic and prognostic information from vast amounts of possible biomarkers by using the results from many studies. In summary, a diagnostic biomarker should provide information about clinical status, and potentially about specific treatment targets. A useful biomarker might also provide the location of the patient’s disorder and the severity of the illness. Similarly, an ideal prognosis biomarker may predict future clinical features, such as the speed of disease progression or the prediction of remission. There are some non-coding RNAs and coding-RNAs (validated as biomarkers) that function as ceRNAs and play an essential role in lung cancer progression. For example, linc81507 acts as a ceRNA for miR-199b-5p by reducing CAV1 expression levels. It indicates the tumor suppressive functions of linc81507 in NSCLC progression, and it can serve as a potential biomarker for diagnosis and prognosis in lung cancer^78^. In addition, NEAT1, a nuclear-enriched abundant transcript1, acts as a ceRNA for hsa-mir-98-5p by directly binding to and interfering with hsa-mir-98-5p-mediated regulation of mitogen-activated protein kinase 6 (MAPK6). It has been suggested as a prognostic biomarker in lung cancer diagnosis^83^. Finally, a novel diagnostic and prognostic biomarker (LncRNA HOTTIP) used for clinical research of lung cancer has been proposed. LncRNA HOTTIP mediates the ceRNA network, which interferes with the expression of anti-apoptotic factor BCL-2, suggesting that HOTTIP may serve as a valuable biomarker in lung cancer patients^90^. However, diagnostic and prognostic biomarkers might have complex correlations with patient outcomes. Biomarkers that indicate a poor prognosis have motivated researchers to seek more effective and favorable therapeutic methods for treatment. The lncRNAs OIP5-AS1 and OGFRP1 that act as ceRNAs have also been found to be effective predictors of advanced clinical stage, lymph node metastasis, and poor prognoses for NSCLC patients, indicating their significant roles in cancer development and progression^46,140^. In summary, the development of diagnostic methods for NSCLC has been slow, and patient prognosis has historically still been regarded as poor. Hence, understanding the intricacies of biomarker interactions and the comprehensive mechanisms of biomarker molecules can increase treatment opportunities for cancer patients and provide more individualized care for patients with a poor prognosis.

Chemotherapy is one of the basic clinical treatments strategies for NSCLC. Cisplatin (DDP) and carboplatin-based chemotherapy drugs are widely used for lung cancer treatment^155^. However, resistance to chemotherapy results in a poor prognosis, and also limits the use of DDP in clinical applications. Chemoresistance has been a major challenge for chemotherapy in human lung cancers. Cisplatin sensitivity is associated with DNA repair, apoptosis, and autophagy, and ceRNAs play essential parts in lung cancer drug resistance^156^. For example, BAG-1 (Bcl-2 associated athanogene-1) is closely related to sensitivity to radio(chemo)therapy. Previous studies have verified that lncRNA XIST acts as a ceRNA to positively regulate the cisplatin resistance of LUAD through the let-7i/BAG-1 axis^84^. Moreover, Beclin-1, a biomarker of autophagy, can inhibit apoptosis and could improve drug resistance. PVT1 might function as a ceRNA for sponging miR-216b and regulating Beclin-1, to enhance cisplatin sensitivity through autophagy and anti-apoptosis85. DNA repair function is a crucial factor that can affect the efficacy of cisplatin resistance. Nucleotide excision repair (NER) is thought to closely determine drug resistance. A study reported that hsa_circ_0001946 might affect the cisplatin sensitivity of lung cancer cisplatin through NER. Checkpoint kinase 1 (CHEK1) is implicated in regulating and identifying DNA damage. It was reported that LINC00485 induced cisplatin resistance through acting as a ceRNA of miR-195 in NSCLC^88^. A combination of etoposide (VP-16)/irinotecan plus cisplatin is a chemotherapy regimen for SCLC. Research results have shown that Linc00173 may function as a ceRNA to affect SCLC chemoresistance improvement via regulating Etk expression^26^. These results also indicate that ceRNAs may serve as novel biomarkers for therapeutic targets and prognostic markers of lung cancer patients.

## Conclusions and perspectives

The roles of non-coding RNA and coding RNA as tumor ceRNAs are currently being investigated. With the development of high-throughput sequencing and bioinformatic analysis technologies, ceRNAs have been found to be involved in many biological functions, including lung cancer generation and development. However, due to complex variable shear events and a limited understanding of the ceRNA mechanisms, the false positive rates of ceRNAs is high, therefore, remaining problems need to be resolved. Recent studies have suggested that dysregulated ceRNAs are involved in many biological processes of lung cancer, including signaling pathways that respond to extracellular cues, cell proliferation, cell apoptosis, cell invasion, and cell migration. In this review, we described recent progress in the field of non-coding and protein-coding RNAs acting as ceRNAs in lung cancer, and highlighted validated ceRNAs involved in lung cancer biological functions. Furthermore, the role of ceRNAs as novel prognostic and diagnostic biomarkers was also reviewed. However, multiple signaling pathways cooperate in the initiation and progression of ceRNA crosstalk in lung cancer, and current knowledge is still incomplete. Hence, further understanding of ceRNA crosstalk control mechanisms in lung cancer and their associated signaling pathways could help to better understand the mechanisms involved in the progression of lung cancer. The roles of pseudogenes acting as ceRNAs to participate in cellular metabolic molecular mechanisms in lung cancer are unclear. Pseudogenes were initially regarded as non-coding RNAs resulting from gene mutations during evolution. Far from being silent, pseudogenes have subsequently been shown to function as ceRNAs that regulate tumor cellular metabolism. Tumor cells express programmed death-ligand 1 (PD-L1), which binds to receptor programmed cell death protein 1 (PD-1). The specific combination of PD-L1/PD-1 can suppress and impair the activation of T cells and enhance the immune tolerance of tumor cells, which eventually could lead to tumor immune escape. Anti-PD-1/PD-L1 immune checkpoint therapy has been recognized as a promising approach for tumor treatment. However, the upstream regulatory mechanism of PD-L1/PD-1 still remains unknown. The circRNA-002178 can promote PD-L1 expression via sponging of miR-34 in lung cancer cells^157^. However, the biological mechanisms by which the ceRNAs competitively target miRNAs to regulate PD-L1/PD-1 in lung cancer are still unclear. Elucidating the precise mechanisms used by ceRNAs to activate or inactive PD-L1 expression is critical for promoting a better understanding of immune escape mechanisms. This knowledge could provide opportunities to identify potential clinical therapeutic targets to improve the efficacy of immunotherapy for lung cancer. An improved understanding of the regulatory mechanisms of ceRNAs may facilitate the development of future clinical applications and their use as prognostic markers or potential therapeutic targets for lung cancer.

However, there are several challenges regarding possible ceRNA therapy. First, the ceRNA regulatory network in lung cancer is multilayered and complicated, and the miRNAs are mainly involved with multiple non-coding RNAs. Therefore, nonspecific manipulation of mutational genes could impact and alter normal gene expressions. Second, an effective and specific delivery vector for ceRNA therapeutic agents must be developed. Ultimately, better therapeutics are needed to develop ceRNA-driven precision medicine, to provide new possibilities and directions for ceRNA use to overcome treatment obstacles, and aid in efforts to isolate and specifically target drugs capable of having anti-tumor activity.

## Abbreviations

CeRNA: competing endogenous RNA; NSCLC: non-small cell lung cancer; SCLC: small cell lung cancer; LUAD: lung adenocarcinoma; LUSC: lung squamous carcinoma; MREs: MiRNA response elements; RBPs: RNA binding proteins; ncRNA: non-coding RNA; lncRNA: long noncoding RNA; HOTAIR: HOX transcript antisense RNA; PDPK1: 3-phosphoinositide-dependent protein kinase-1; TTN-AS1: Titin-antisense RNA1; CDK5: cyclin dependent kinase 5; EMT: epithelial-mesenchymal transition; CBS: cystathionine-β-synthase; LCAT1: lung cancer associated transcript 1; RAC1: Rac family small GTPase 1; TINCR: terminal differentiation-induced lncRNA; UCA1: urothelial carcinoma associated 1; PVT1: plasmacytoma variant translocation 1; MMP2: matrix metalloproteinase 2; XIAP: X-linked inhibitor of apoptosis; HOTTIP: HOXA transcript at the distal tip; EZH1: enhancer of zeste homolog 1; KLK4: Kallikrein-related peptidase 4; FAIM2: Fas apoptosis inhibitory molecule 2; ZEB1: Zinc-finger E-box binding homeobox 1; ZEB1-AS1: ZEB1 antisense RNA 1; NEAT-1: nuclear enriched abundant transcript 1; E2F3: E2F transcription factor 3; PRNCR1: prostate cancer non-coding RNA 1; HEY2: hairy and enhancer of split-related with YRPW motif protein 2; ADAM 15: a disintegrin and a metalloproteinase 15; PLSCR4: phospholipid scramblase; TP73-AS1: lncRNA antisense RNA of the TP73 gene; XIST: X-inactive specific transcript; PXN: Paxillin; TGF-β: transforming growth factor-β; TGFBR2: TGF-β receptor type II; MYEOV: myeloma overexpressed gene; USP15: ubiquitin specific protease 15; MALAT1: metastasis-associated lung adenocarcinoma transcript 1; TFAP2C: transcription factor AP-2 gamma; HOXD-AS1: HOXD Cluster Antisense RNA; DANCR: differentiation antagonizing noncoding RNA; DGCR5: DiGeorge syndrome critical region gene 5; EPHB6: EPH receptor B6; SNHG20: small nucleolar RNA host gene 20; DLX6-AS1: distal-less homeobox 6 antisense 1; PRR11: proline rich 11; SNHG6: small nucleolar RNA host gene 6; mTOR: mammalian target of rapamycin; RRM2: ribonucleotide reductase M2 subunit; SNHG1: small nucleolar RNA host gene 1; MTDH: metadherin; IGF-2: insulin-like growth factor-2; CAV1: caveolin1; NORAD: non-coding RNA activated by DNA damage; MINCR: MYC induced long noncoding RNA; MINCR: MYC induced long noncoding RNA; CCND2: cyclin D2; Bcl-2: B-cell lymphoma-2; Bax: BCL2-associated X; MAPK6: mitogen-activated protein kinase 6; MRP1: multidrug resistance protein 1; CHEK1: checkpoint kinase 1; ZFAS1: ZNFX1 antisense RNA1; EGF: epidermal growth factor; EGFR: epidermal growth factor receptor; DUSP1: dual-specificity phosphatase 1; FOXO1: forkhead box O1; RTKs: ectopic expression of receptor tyrosine kinases; PPDPF: Pancreatic progenitor cell differentiation and proliferation factor; MACC1: metastasis-associated in colon cancer 1; STAT3: signal transducer and activator of transcription 3; STK11: serine/threonine kinase 11; ENO1: enolase 1; cytC: cytochrome C; XIAP: Xlinked inhibitor of apoptosis protein; BRD4: bromodomain-containing protein 4 (BRD4) ; MADD: MAPK-activating death domain-containing protein; cESRP1: circular RNA epithelial splicing regulatory protein-1; TGFβR1: TGF-β receptor type I; PTEN: phosphatase and tensin homolog; MMPs: matrix metalloproteases; ERK: extracellular regulated kinase; MEK: mitogen-activated protein kinase/Erk kinase; E-cadherin: epithelial cadherin; GSK3β: glycogen synthase kinase-3β.
